# CRITICAL APPRAISAL OF THE CLINICAL TRIAL: EFFECT OF COLONOSCOPY SCREENING ON RISKS OF COLORECTAL CANCER AND RELATED DEATH

**DOI:** 10.1590/0102-672020230002e1719

**Published:** 2023-03-20

**Authors:** Wanderley Marques Bernardo, Marcelo Averbach, Eduardo Guimarães Hourneaux de Moura

**Affiliations:** 1 Universidade de São Paulo, Faculty of Medicine, Clinical Hospital, Program of the Gastrointestinal Endoscopy Service, Departament of Gastroenterology – São Paulo (SP), Brazil.

First, we would like to congratulate the authors of this study^1^ for stimulating reflection on public health policy involving the diagnosis of colorectal cancer (CRC), a topic of extreme relevance, given that it is the third most common cancer in men and the second most common cancer in women. In 2020, there were more than 1.9 million new cases of CRC^6^.

The benefits of CRC screening were recognized four decades ago when the American Cancer Society started recommending it. The screening was responsible for the decline in CRC incidence observed since the 1980s^2^.

Randomized studies have shown that screening people at medium risk, that is, those with no family history of CRC, reduces the incidence and mortality resulting from this neoplasm^2,3^.

In contrast to screening programs for other neoplasms, CRC screening allows the diagnosis of lesions at an early stage and the detection of pre-malignant lesions that, if removed, can prevent cancer^4^.

The importance of CRC screening is based not only on the possibility of early diagnosis but also on the impact of endoscopic polypectomy, which reduces mortality related to this neoplasm by more than 50%^7^.

The comments below discuss the methodology used in the study (NordiCC Study) and the negative impact on a diagnostic technique established in several publications due to structural errors added to the article.

Pragmatic (real-life) randomized controlled trials (RCTs) can be considered observational studies (cohorts) in which, although randomization is present, it does not give these trials the character of experimentation (associated with the term randomized clinical trial) – the rigorous individual eligibility criteria are a fundamental part of the methodology. Even when these eligibility criteria are “relaxed,” the analysis in these (pragmatic) trials must necessarily consider prognostic differences between participants, which are essential to avoid confounding and selection bias. In addition, the term pragmatic may be misused as the cohort departs from usual practice because, despite the randomization of participants being performed at the group level, the proposed interventions do not correspond to the conventional care that these patients would receive, as an invitation for colonoscopy, periodic contact, or even the creation of a control group without care (called usual care). Also, the absence of care in the usual care group (comparison) calls into question the classic concept of randomization since there is no control in this group regarding losses or migration (crossover) to colonoscopy.

Intention-to-treat analysis is prohibitive due to the extensive loss of adherence (non-compliers) of participants in the invited (screened) group, decreasing the sample in this group after randomization by more than 50%. The only possible analysis is per protocol. Furthermore, non-complier patients (who did not accept the invitation) and conventional care patients should be analyzed within the same group (not screened) and compared with participants who actually underwent colonoscopy.

## ANALYSIS

**1. Per protocol**=no screening (**56,365**) vs. compliers (**11,843**)

### Cancer risk difference (PPP)

Cancer risk screening (PPP): 102/11,843=0.86%

Cancer risk usual care (PPP): 622/56,365=1.1%

Cancer risk difference (PPP)=0.24% (95%CI, 0.42–0.05)

Number need to screen and diagnose cancer=416

### Difference in risk of death (PPP)

Risk of death screening (PPP): 17/11,843=0.14%

Risk of death usual care (PPP): 157/56,365=0.27%

Death risk difference (PPP)=0.13% (95%CI 0.21–0.05%)

Number need to screen and prevent death=769

**2. Per protocol aggregated (non-compliers + usual care) vs. compliers**

Non-compliers (**16,377**) + usual care (**56,365**)=72,742 vs. compliers (**11,843**)

### Cancer risk difference (PPP aggregate)

Cancer risk screening (PPP aggregate): 102/11,843=0.86%

Cancer risk usual care (PPP aggregate): 779/72,742=1.0%

Cancer risk difference (PPP aggregate)=0.20% (95%CI, 0.39–0.02)

Number need to screen and diagnose cancer=500

### Risk of death difference (control)

Risk of death screening (control): 17/11,843=0.14%

Death risk usual care (control): 212/72,742=0.29%

Death risk difference (control)=0.14% (95%CI 0.22–0.06)

Number need to screen and prevent death=714

### Main critical points

Risk of bias very high

- Patient selection bias (absence of prognostic similarity between the two compared groups).
- Confounding bias (uncertainty of absence of baseline outcome–particularly in conventional care patients)
- Absence of blinding and losses greater than 20%
- Sample size calculation based on “inflated” estimates of a 25% cancer mortality difference between screened and unscreened
- The sample size calculation was estimated for 15 years of follow-up, and this publication is characterized by preliminary results or “interim analysis” or early discontinuation (loss of blinding).
- The mean follow-up of 10 years should not be used, and only patients already followed up for a period of at least 10 years should have been considered in the analysis, that is, from 2009 to 2011.
- Screening only those patients who accept the invitation can select a group of patients with prognostic factors or characteristics, which must be different from the other participants (who did not accept the invitation) and may favor the diagnosis (diagnostic bias).
- The number of events (mortality and cancer incidence) is very small, giving uncertainty to the differences obtained.

## CONCLUSION

The NordICC pragmatic study has serious methodological limitations, resulting in high uncertainty. However, in the per-protocol analyses, whether or not aggregating non-compliers patients to those in usual care, it is demonstrated that:

**PPP**: 0.24% increase in colon cancer diagnosis with screening, requiring screening of 416 patients to diagnose colon cancer. Reduction in risk of death of 0.13% with screening, requiring screening of 769 patients to avoid one death from colon cancer.

**Aggregate PPP**: 0.20% increase in colon cancer diagnosis with screening, requiring screening of 500 patients to diagnose colon cancer. Reduction in risk of death of 0.14% with screening, requiring screening of 714 patients to avoid one death from colon cancer.

The data obtained in the published article and in the supplement demonstrate colonoscopy’s substantial benefit.

Comparing with breast cancer screening, we can cite a meta-analysis of observational studies that indicates a relative risk (RR) of mortality for breast cancer of 0.86 for those patients with age between 50 and 59 years (95%CI 0.68–0.97), with 8 deaths being avoided per 10,000 women in 10 years and 0.67 for those aged 60–69 years (95%CI 0.54–0.83), preventing 21 deaths per 10,000 women in 10 years^2,5^. The role of mammography in breast cancer screening is clearly evidenced, and this policy is consecrated. These results are very similar to colon cancer screening by colonoscopy.

We respectfully suggest that the editors of the *New England Journal of Medicine* re-do the statistical analyses, confirm our observations, and claim redress, given the negative impact of this study on CRC screening.

## References

[1] Bretthauer M, Løberg M, Wieszczy P, Kalager M, Emilsson L, Garborg K (2022). Effect of colonoscopy screening on risks of colorectal cancer and related death. N Engl J Med.

[2] Hardcastle JD, Chamberlain JO, Robinson MH, Moss SM, Amar SS, Balfour TW (1996). Randomised controlled trial of faecal-occult-blood screening for colorectal cancer. Lancet.

[3] Holme Ø, Løberg M, Kalager M, Bretthauer M, Hernán MA, Aas E (2014). Effect of flexible sigmoidoscopy screening on colorectal cancer incidence and mortality: a randomized clinical trial. JAMA.

[4] Lieberman D, Ladabaum U, Cruz-Correa M, Ginsburg C, Inadomi JM, Kim LS (2016). Screening for colorectal cancer and evolving issues for physicians and patients: a review. JAMA.

[5] Nelson HD, Fu R, Cantor A, Pappas M, Daeges M, Humphrey L (2016). Effectiveness of breast cancer screening: systematic review and meta-analysis to update the 2009 U.S. Preventive Services Task Force Recommendation. Ann Intern Med.

[6] World cancer research fund international Colorectal cancer statistics.

[7] Zauber AG, Winawer SJ, O’Brien MJ, Lansdorp-Vogelaar I, van Ballegooijen M, Hankey BF (2012). Colonoscopic polypectomy and long-term prevention of colorectal-cancer deaths. N Engl J Med.

